# Drug-Resistant Tuberculous Spondylitis Treated with Bedaquiline-Containing Regimens in South Korea: Two Case Reports

**DOI:** 10.3390/antibiotics15050493

**Published:** 2026-05-14

**Authors:** Keon Young Lee, Miri Hyun, Ji Yeon Lee, Hyun ah Kim

**Affiliations:** Department of Infectious Diseases, Keimyung University Dongsan Hospital, Keimyung University School of Medicine, Keimyung University, Daegu 42601, Republic of Korea; lkyoung121@dsmc.or.kr (K.Y.L.); eternity7919@dsmc.or.kr (M.H.); jirong84@dsmc.or.kr (J.Y.L.)

**Keywords:** drug-resistant tuberculosis, extrapulmonary tuberculosis, tuberculous spondylitis, bedaquiline

## Abstract

**Background:** South Korea continues to report a considerable burden of drug-resistant tuberculosis (TB). Bedaquiline-containing regimens are recommended for multidrug-resistant pulmonary TB, but evidence regarding the optimal treatment for extrapulmonary manifestations such as spinal TB remains limited. **Case presentation:** Herein, we report two cases of drug-resistant tuberculous spondylitis that were successfully managed using bedaquiline-containing regimens. Case 1 involved a 67-year-old man who was receiving chemotherapy for lymphoma and had a history of spinal TB treated 20 years earlier. The patient presented with dysphagia and upper limb weakness. Cervical magnetic resonance imaging revealed C4–5 spondylitis with an epidural abscess. He underwent surgical treatment, and *Mycobacterium tuberculosis* resistant to rifampin was isolated from cultured intraoperatively obtained tissue specimens. The patient received an antibiotic regimen consisting of bedaquiline, levofloxacin, linezolid, cycloserine, and clofazimine. Clinical and radiological improvements were achieved after 12 months of this treatment; bedaquiline was included in the regimen for the first 6 months, while the other agents were continued for the entire course. Case 2 involved a 71-year-old man with T12–L2 spondylitis and a left psoas abscess. Tissue culture confirmed *Mycobacterium tuberculosis* resistant to isoniazid, rifampin, and ethambutol. The patient was started on the same bedaquiline-containing regimen. Clinical and radiological improvements were observed after 18 months of this therapy, including 6 months of bedaquiline. **Conclusions:** Our clinical experiences suggest that bedaquiline-containing regimens represent a feasible and effective therapeutic option for drug-resistant tuberculous spondylitis. Larger studies are warranted to establish the optimal management strategies for extrapulmonary drug-resistant TB infections.

## 1. Introduction

Tuberculous spondylitis is the most common form of skeletal tuberculosis (TB) among the extrapulmonary types of TB infection, accounting for ~10% of all extrapulmonary TB infection and nearly half of osteoarticular TB [1]. This condition poses significant clinical challenges because of its potential to cause severe complications such as vertebral destruction, neurological deficits, and spinal deformities [2]. Early diagnosis and appropriate treatment are therefore crucial to preventing irreversible morbidity and mortality associated with tuberculous spondylitis [3].

Multidrug-resistant (MDR)-TB and extensively drug-resistant (XDR)-TB have also been identified in patients with extrapulmonary TB, including tuberculous spondylitis [4]. The prevalence of MDR-TB and XDR-TB in spinal TB is comparable to that observed in pulmonary TB and varies by geographic region, having been reported to range from 11 to 30% in patients with culture-positive tuberculous spondylitis [4,5]. The management of drug-resistant tuberculous spondylitis presents considerable therapeutic challenges. These infections typically require prolonged treatment courses lasting 18–24 months or longer, with substantially higher treatment failure rates than those of drug-susceptible TB [6].

Bedaquiline, a diarylquinoline-class anti-tuberculous agent, represents a key therapeutic option with a unique mechanism of action that is distinct from those of conventional anti-TB drugs [7]. By inhibiting mycobacterial adenosine triphosphate (ATP) synthase and disrupting cellular energy metabolism in *Mycobacterium tuberculosis*, bedaquiline offers superior efficacy for the treatment of both MDR-TB and XDR-TB [8]. Bedaquiline is now a core component of a shorter, 6-month, all-oral regimen for MDR and rifampin-resistant (RR) TB, consisting of bedaquiline, pretomanid, linezolid, and moxifloxacin (BPaLM), which has demonstrated efficacy and acceptable safety profiles in multiple global cohorts [9,10]. Although bedaquiline has been primarily used to treat pulmonary TB, its usage for extrapulmonary TB, particularly in spinal MDR-TB, has not been well studied [6]. One Indian study that evaluated bedaquiline and delamanid therapies in patients with drug-resistant extrapulmonary TB, including two cases of spinal TB, found that bedaquiline- and delamanid-based regimens were effective with minimal adverse events [11]. A recent Italian case report described successful treatment outcomes using bedaquiline-containing regimens in patients with MDR-TB spinal osteomyelitis and soft tissue abscesses, suggesting the potential effectiveness of this approach for drug-resistant tuberculous spondylitis as well [12].

South Korea has one of the highest incidence rates of TB among Organization for Economic Co-operation and Development (OECD) nations, making the management of drug-resistant TB a particularly crucial public health priority in the region [13,14]. A recent South Korean epidemiological study reported that MDR/RR-TB accounted for 2.4% of the extrapulmonary TB cases identified [15]. However, published reports concerning the treatment of drug-resistant tuberculous spondylitis using bedaquiline-containing regimens in South Korea remain extremely limited in the literature. Herein, we report two cases of drug-resistant tuberculous spondylitis that were successfully treated using bedaquiline-containing regimens at a tertiary hospital in South Korea.

## 2. Case Presentation

### 2.1. Case 1

#### 2.1.1. Clinical Presentation, Radiological Findings, and Initial Diagnostic Confusion

A 67-year-old man with a complex medical history presented to our hospital with dysphagia in July 2022. His comorbidities included diffuse large B-cell lymphoma of the scalp, for which he was undergoing chemotherapy at the time, prior tuberculous spondylitis that had been treated in 1996, diabetes mellitus, hypertension, systemic lupus erythematosus with polymyositis, and non-ST elevation myocardial infarction.

Laboratory analysis on admission demonstrated elevated inflammatory markers, with a white blood cell (WBC) count of 21,020/μL (71.7% neutrophils) and a C-reactive protein (CRP) level of 5.3 mg/dL.

**Figure 1:**
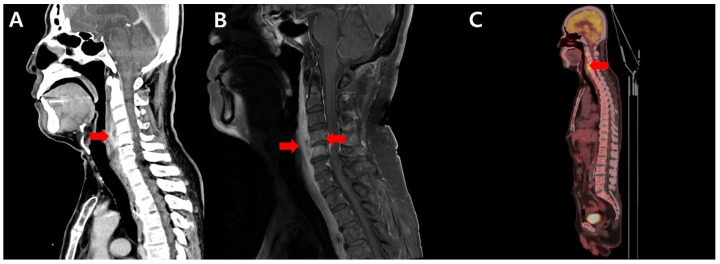
Initial diagnostic imaging of tuberculous spondylitis: (**A**) Enhanced neck CT demonstrating a hypoattenuated lesion at the C4–5 level with prevertebral soft tissue involvement (red arrow). (**B**) T1-weighted fat-suppressed sagittal cervical spine MRI showing C4–5 disco-vertebral destruction with prevertebral and epidural infiltrations (red arrows) causing moderate to severe cord compression. (**C**) PET-CT demonstrating intense FDG uptake around the C4–C5 vertebral bodies and diffuse hyperplastic bone marrow FDG uptake (red arrow).

An imaging study was performed to evaluate the patient’s dysphagia. Neck computed tomography (CT) confirmed a hypoattenuated lesion at the C4–5 level with prevertebral soft tissue involvement (Figure 1A). Enhanced cervical spinal magnetic resonance imaging (MRI) revealed diffuse mild bone marrow enhancement, prominent enhancing infiltration of the prevertebral soft tissue, extensive peripheral edema at the C5 level, and a focal cavity-like lesion. Collectively, these findings suggested infectious spondylodiscitis (Figure 1B). Positron emission tomography (PET)-CT was performed for the differential diagnosis of lymphoma. This revealed newly formed intense fluorodeoxyglucose (FDG) uptake around the C4 and C5 vertebral bodies and diffuse FDG uptake in the bone marrow with a hyperplastic appearance, requiring a differential diagnosis among possible compression fracture, spondylitis, and lymphoma involvement (Figure 1C).

Our microbiological investigations initially caused some diagnostic confusion. Given the patient’s neurological symptom of dysphagia, the neurosurgery department recommended surgical decompression; however, the patient declined both surgery and localized spinal needle biopsy because of his underlying malignancy. Because *Staphylococcus lugdunensis* (*S. lugdunensis*) was isolated from the patient’s initial central line blood culture, the initial clinical impression was pyogenic spondylitis. The isolate was susceptible to all tested antimicrobial agents, including benzylpenicillin, linezolid, and vancomycin.

#### 2.1.2. Initial Empiric Treatment and Clinical Deterioration

Given the isolation of methicillin-susceptible *S*. *lugdunensis* from the initial blood culture and the clinical and radiologic impression of pyogenic spondylitis, empirical intravenous cefazolin was initiated. After 2 weeks of cefazolin therapy, the patient demonstrated clinical stability with improvement in inflammatory markers, including a WBC count of 5730/μL (31.1% neutrophils) and a CRP level of 0.4 mg/dL. The antibiotic regimen was subsequently changed to oral cephalexin, and the patient was discharged.

**Figure 2:**
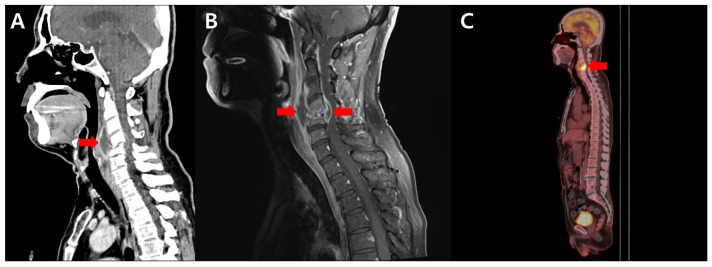
Follow-up preoperative progression and diagnostic confirmation. (**A**) Enhanced neck CT confirming an ~4 cm hypo-attenuated prevertebral abscess with rim enhancement at the C3–5 level, causing severe cord compression (red arrow). (**B**) T1-weighted fat-suppressed sagittal cervical spine MRI showing significant progression with enlarged prevertebral and epidural abscesses (red arrows), increased bone marrow infiltration, and a C5 compression fracture, despite the ongoing cephalexin therapy. (**C**) PET-CT showing diffuse FDG uptake in the C4–5 vertebral bodies and prevertebral space, with a compression fracture at the C5 level (red arrow).

However, 1 month after his discharge, while continuing the cephalexin therapy as an outpatient, the patient developed worsening shoulder pain with left shoulder abduction and elbow flexion weakness (grade 2–3). Laboratory analysis demonstrated an elevated WBC count of 13,760/μL (70% neutrophils), excess CRP level of 4.7 mg/dL, and erythrocyte sedimentation rate (ESR) of 113 mm/h. Neck CT confirmed an ~4 cm hypo-attenuated lesion with rim enhancement at the C3–5, with a compression fracture at C5 (Figure 2A). Repeated enhanced spinal MRI revealed the progression of infectious spondylodiscitis with markedly increased destruction of the disco-vertebral junction, as well as prevertebral and epidural abscess formation at the C4–5 level (Figure 2B). PET-CT revealed diffuse FDG uptake in the C4–5 vertebral bodies and prevertebral spae, with a compression fracture at C5 (Figure 2C).

#### 2.1.3. Surgical Intervention and Mycobacterial Discovery

Given the progressive neurological deterioration and radiological progression, despite the appropriate ongoing antibiotic therapy for the patient’s *S. lugdunensis* infection, urgent anterior cervical discectomy and fusion with autologous iliac bone grafting at C4–5 was performed. Extensive yellowish purulent material was encountered intraoperatively. The prevertebral fascia was opened, revealing large quantities of pus that were sampled for culturing. Significant prevertebral and epidural abscesses were identified with granulation tissue formation, which was carefully debrided.

Intraoperative pus and tissue specimens were submitted for bacterial, fungal, and mycobacterial cultures. Bacterial and fungal culture results were negative. Acid-fast bacillus (AFB) staining was negative, but polymerase chain reaction (PCR) testing for *Mycobacterium tuberculosis* returned positive results. Biopsy revealed granulomatous inflammation with necrotic centers, and real-time TB PCR was positive, which is pathognomonic for TB infections.

#### 2.1.4. Drug Susceptibility Testing and Anti-TB Therapy with a Bedaquiline-Containing Regimen

Anti-TB therapy was empirically initiated with isoniazid, rifampin, ethambutol, and pyrazinamide (the “HERZ” regimen). Levofloxacin was also added, based on our postoperative findings, the patient’s prior history of tuberculous spondylitis, and our team’s clinical suspicion. Approximately 6 weeks after the initial surgery, mycobacterial culture of the original intraoperative specimen yielded *Mycobacterium tuberculosis*, and subsequent drug susceptibility testing on the cultured isolate identified RR-*Mycobacterium tuberculosis*. Tissue specimens obtained intraoperatively were cultured on an Ogawa solid medium and a mycobacteria growth indicator tube (MGIT) liquid medium. The genotypic drug susceptibility test (DST) was conducted by the Xpert MTB/RIF assay, and the phenotypic DST was performed by the absolute-concentration method on a Lowenstein–Jensen medium.

Genotypic DST of the intraoperative sample revealed rifampin resistance with the specific *rpoB* S531L mutation. Phenotypic DST of the same specimen demonstrated rifampin and rifabutin resistance. However, the specimen was susceptible to all of the other first- and second-line agents tested, including pyrazinamide, ethambutol, levofloxacin, and moxifloxacin. This resistance pattern is known as RR-TB.

The first-line HERZ regimen was discontinued and replaced with levofloxacin, linezolid, cycloserine, ethambutol, and pyrazinamide. Following approval by the national MDR-TB committee, a bedaquiline-containing regimen was initiated, which consisted of bedaquiline (400 mg daily for 2 weeks, followed by 200 mg three times weekly), along with levofloxacin, linezolid, cycloserine, and clofazimine. Bedaquiline was continued for the planned 6-month duration. After this bedaquiline course was completed, the levofloxacin, cycloserine, and clofazimine were continued until 18 December 2023.

#### 2.1.5. Treatment Response and Clinical Outcome

**Figure 3:**
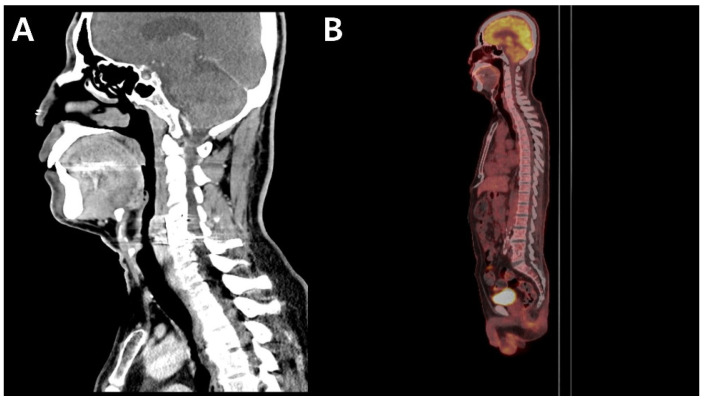
Follow-up imaging demonstrating the patient’s clinical outcome following the bedaquiline-containing anti-tuberculosis therapy: (**A**) Follow-up neck CT demonstrating resolution of the prevertebral and epidural abscesses. (**B**) Follow-up PET-CT demonstrating complete disappearance of FDG uptake in the vertebral bodies and prevertebral space.

Follow-up imaging revealed a marked radiological response to the anti-TB therapy. Follow-up neck CT performed in March 2023 demonstrated resolution of the prevertebral and epidural abscesses (Figure 3A). Compared with the baseline PET-CT from September 2022, the follow-up PET-CT in June 2023 demonstrated complete disappearance of FDG uptake in the prevertebral space, indicating substantial resolution of the inflammatory and infectious processes (Figure 3B).

The patient demonstrated good adherence to the prolonged anti-TB regimen throughout the treatment course. Adherence was monitored at each outpatient visit by self-report, pill counts, and clinical response, and no doses were reported to have been missed. Upon completion of the anti-TB therapy in December 2023, the patient demonstrated substantial clinical improvement. His neurological symptoms, including bilateral shoulder pain and motor weakness, had resolved entirely. Laboratory analysis at treatment completion showed improvements in his inflammatory marker levels, with a CRP level of 0.4 mg/dL and an ESR of 48 mm/h.

### 2.2. Case 2

#### 2.2.1. Clinical Presentation and Initial Diagnosis

**Figure 4:**
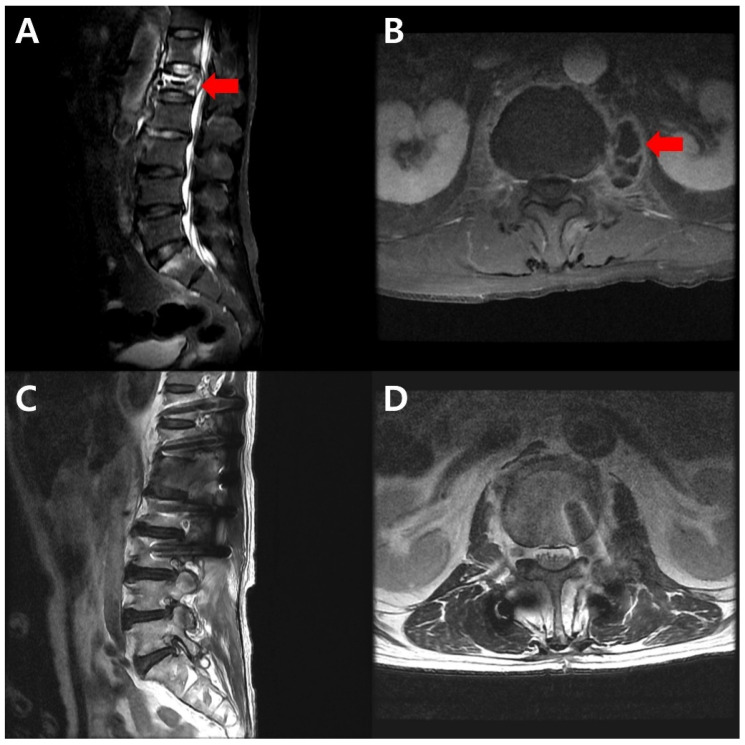
Spinal MRI findings with thoracolumbar tuberculous spondylitis before and at the end of the treatment. (**A**) A T2-weighted sagittal sequence in the baseline spine MRI revealed destruction of the vertebral bodies, particularly at L1, with contrast enhancement (red arrow). (**B**) A T1-weighted fat-suppressed axial sequence in the baseline spine MRI demonstrated a left psoas abscess (red arrow). (**C**) A T2-weighted sagittal sequence in the follow-up MRI obtained at the end of the therapy demonstrated marked resolution of the inflammatory changes in the vertebral body. (**D**) A T2-weighted axial sequence in the follow-up MRI obtained at the end of the therapy demonstrated improvement of the left psoas abscess.

A 71-year-old male patient with significant medical comorbidities, including atrial fibrillation, myocardial infarction, and a history of gastric ulcer bleeding, presented to our institution in June 2023 for a secondary opinion regarding the management of MDR-TB spondylitis. The patient had initially presented to a referring hospital with complaints of back pain. Enhanced spine MRI revealed destruction of the T12–L2 vertebral bodies and a left psoas abscess, which were considered consistent with spondylitis (Figure 4A,B).

#### 2.2.2. Surgical Intervention and Microbiological Findings

In April 2023, the patient underwent a comprehensive surgical intervention consisting of anterior corpectomy at L1, anterior fusion at T12–L2, and posterior fixation extending from T11 to L3. Intraoperative tissue specimens were obtained and sent for culturing and histopathological examination. Initial microscopic examination revealed trace AFB, and PCR testing confirmed the presence of *Mycobacterium tuberculosis*.

Histopathological analysis of the tissue specimens showed chronic granulomatous inflammation characterized by collections of epithelioid histocytes with scattered multinucleated giant cells, central caseous necrosis, and positive AFB staining, further indicating TB infection. As in Case 1, tissue specimens obtained intraoperatively were cultured on an Ogawa solid medium and a MGIT liquid medium. Intraoperative tissue cultures grew *Mycobacterium tuberculosis*. The genotypic DST was performed using the Xpert MTB/RIF assay, and the phenotypic DST was conducted using the absolute-concentration method on a Lowenstein–Jensen medium.

Genotypic DST performed in May 2023 demonstrated resistance to both rifampin and isoniazid. Molecular analysis revealed resistance-associated mutations, including loss of the *rpoB* 509–514 probe and *katG* S315T mutation. Subsequent phenotypic DST revealed resistance to rifampin, isoniazid, ethambutol, and rifabutin. The patient was referred to our hospital in June 2023 to undergo specialized management of MDR-TB spondylitis.

#### 2.2.3. Treatment Course and Clinical Outcome

At the referring institution, the patient was initially started on a standard HERZ first-line anti-TB therapy. Following the identification of rifampin and isoniazid resistance in May 2023, this regimen was modified to include ethambutol, pyrazinamide, moxifloxacin, and intravenous amikacin.

Upon referral to our institution in June 2023, initial laboratory findings revealed a WBC count of 6700/μL (73.2% neutrophils) and a CRP level of 3.4 mg/dL. Following approval by the national MDR-TB committee, a regimen consisting of bedaquiline, linezolid, cycloserine, clofazimine, and levofloxacin was administered.

During the course of treatment in October 2023, after ~4 months of therapy, the patient developed peripheral neuropathy with numbness in the right lower extremity. Consequently, linezolid was discontinued, and pyrazinamide was added to the regimen. Bedaquiline was discontinued after 6 months, after which a four-drug regimen was maintained for the remaining treatment period.

Clofazimine was discontinued in June 2024, because the patient developed significant skin hyperpigmentation and poor oral intake. A three-drug regimen was then maintained for an additional 6 months, concluding the anti-TB treatment course.

Upon completion of the anti-TB therapy, laboratory findings demonstrated substantial improvement. The final laboratory findings in December 2024 showed a WBC count of 8380/μL (67.3% neutrophils), CRP level of 0.5 mg/dL, and ESR of 9 mm/h. Follow-up spinal MRI demonstrated marked resolution of the inflammatory changes and significant improvement of the left psoas abscess (Figure 4C,D). The patient’s clinical symptom of back pain also showed considerable improvement, correlating with the radiological findings.

## 3. Discussion

These two cases illustrate the clinical complexity of drug-resistant tuberculous spondylitis and highlight the potential utility of bedaquiline-containing regimens for treating them in the South Korean setting. Despite significant diagnostic and therapeutic challenges, both patients in our series achieved favorable clinical, laboratory, and radiological outcomes.

Establishing a timely and accurate diagnosis of tuberculous spondylitis remains challenging. A definitive diagnosis requires tissue acquisition; however, the diagnostic yield of mycobacterial culture and staining from tissue specimens is limited, and differentiation from pyogenic spondylitis is often challenging based on clinical and radiological findings alone [16]. In Case 1, the concurrent isolation of *S*. *lugdunensis* from the patient’s blood cultures led to an initial suggestion of pyogenic spondylitis, which delayed recognition of the underlying tuberculous etiology. This case underscores the importance of maintaining a high index of suspicion for tuberculous spondylitis when clinical improvement is not achieved with standard antibacterial therapies, particularly in immunocompromised patients or those with a prior history of TB.

In regions where TB is endemic, clinicians should maintain a high index of suspicion for psoas TB when patients present with a psoas abscess [17]. Early recognition is essential to initiate appropriate anti-TB therapy promptly, thereby preventing unwarranted surgical procedures and their associated complications [18]. Furthermore, psoas TB may occur through direct extension from neighboring tuberculous foci in the spine or joints, particularly when there is rupture or erosion of paravertebral cold abscess walls, emphasizing the importance of comprehensive imaging evaluation in suspected cases [19].

The two cases presented here demonstrated distinct but clinically relevant resistance profiles. Case 1 was classified as RR-TB, whereas Case 2 fulfilled the criteria for MDR-TB, defined as resistance to both rifampin and isoniazid [6]. Genotypic DST in Case 1 identified the *rpoB* S531L mutation, one of the most common rifampin resistance-conferring mutations, accounting for ~40–70% of rifampin-resistant clinical isolates globally [20]. In Case 2, genotypic DST revealed loss of the *rpoB* 509–514 probe region, along with the *katG* S315T mutation. The latter is the most common isoniazid resistance-associated mutation, present in ~50–90% of isoniazid-resistant isolates [21,22,23]. In both cases, rapid genotypic resistance detection enabled timely regimen modification without the delays associated with culture-based phenotypic results, facilitating an early transition to an appropriate second-line regimen that included bedaquiline. However, the genotypic DST still required prior isolation of *Mycobacterium tuberculosis* in a mycobacterial culture, and the slow-growing nature of the organism resulted in a diagnostic interval of approximately 6 weeks before a complete resistance profile became available in both cases. Such a delay underscores the need for adjunctive strategies to shorten the time to comprehensive resistance characterization, such as the broader implementation of rapid molecular assays applied directly to clinical specimens or targeted next-generation sequencing performed directly on primary specimens [24,25].

Bedaquiline exerts its antimycobacterial activity through selective inhibition of mycobacterial F-ATP synthase, specifically targeting the c-subunit of the enzyme’s rotor ring [26,27]. Notably, bedaquiline demonstrates bactericidal activity against both actively replicating and non-replicating mycobacterial populations, including metabolically dormant bacilli that could cause treatment relapse [28,29]. The efficacy of bedaquiline in MDR/RR-TB has been established through clinical trials evaluating the BPaLM regimen—comprising bedaquiline, pretomanid, linezolid, and moxifloxacin—which demonstrated noninferiority to conventional longer MDR-TB treatment regimens in the ZeNix and TB-PRACTECAL trials, with treatment success rates exceeding 85% at 6 months [9,30]. These findings support World Health Organization (WHO)-consolidated guidelines, which endorsed BPaLM as the preferred shorter regimen for treating MDR/RR-TB [31]. However, the evidence underlying these recommendations was derived predominantly from trials that enrolled patients with pulmonary TB. Therefore, the direct applicability of these findings to extrapulmonary diseases, particularly skeletal or spinal TB, remains uncertain. In our two cases, bedaquiline was administered for 6 months in line with the duration recommended for pulmonary TB, while the remaining companion anti-TB drugs were continued thereafter. Both patients achieved favorable clinical outcomes. However, evidence to guide the optimal duration of bedaquiline therapy in extrapulmonary TB, particularly spinal TB, is still lacking. Further studies are warranted to establish the appropriate duration of bedaquiline therapy for patients with spinal TB.

The clinical use of bedaquiline also requires careful consideration of its safety profile, tolerability, and impact on patient compliance. Prolongation of the corrected QT interval is the most clinically significant adverse effect and necessitates baseline and serial electrocardiographic monitoring, particularly when bedaquiline is co-administered with other QT-prolonging agents [32]. Hepatotoxicity, nausea, arthralgia, and headache have also been reported [7]. Beyond bedaquiline-specific considerations, multidrug regimens for drug-resistant TB are characterized by prolonged treatment duration, substantial pill burden, and overlapping toxicities, all of which can compromise patient adherence [33]. These considerations highlight the importance of close clinical and laboratory monitoring, active management of adverse events, and structured adherence support throughout the treatment course.

Evidence supporting the use of bedaquiline to treat osteoarticular and spinal TB remains limited to small observational studies and case reports [11,12]. Although formal pharmacokinetic data for spinal tissue are lacking, bedaquiline is characterized by extensive tissue distribution and a prolonged terminal half-life exceeding 5 months [34]. Our present findings suggest that adequate concentrations of bedaquiline may be achievable even at sites of skeletal TB infection. However, further pharmacokinetic studies evaluating the penetration of the drug into bones, intervertebral disks, and paraspinal abscess fluid are warranted to establish a pharmacokinetic–pharmacodynamic rationale for its use in skeletal TB.

A critical gap in the current management of drug-resistant tuberculous spondylitis is the absence of disease-specific, evidence-based treatment guidance. The current WHO and national TB program guidelines for MDR/RR-TB were developed largely on the basis of pulmonary TB data and therefore do not fully address issues specific to spinal TB, including tissue drug penetration, treatment duration, the role and timing of surgical intervention, and optimal strategies for monitoring response [31]. In the present cases, bedaquiline was administered for the standard 6-month course consistent with pulmonary MDR-TB protocols; however, whether an extended bedaquiline regimen might confer additional benefits for deep-seated skeletal infections remains unknown. Future prospective evaluations of bedaquiline-containing regimens for extrapulmonary drug-resistant TB through multicenter observational studies are therefore warranted.

## 4. Conclusions

We recently encountered two cases of drug-resistant tuberculous spondylitis that we successfully treated with bedaquiline-containing regimens at a tertiary hospital in South Korea. Our findings contribute to the limited but growing body of evidence supporting the incorporation of bedaquiline into treatment regimens for drug-resistant extrapulmonary TB and suggest that bedaquiline-containing multidrug regimens represent a viable and effective therapeutic strategy for spinal MDR/RR-TB. Given the continued burden of drug-resistant TB in South Korea, as well as globally, and the current absence of disease-specific treatment guidelines for drug-resistant extrapulmonary TB, prospective multicenter studies that systematically evaluate the efficacy, safety, optimal regimen, and treatment duration of bedaquiline-containing regimens for treating spinal, as well as other forms of extrapulmonary drug-resistant TB, are warranted.

## Data Availability

The original contributions presented in this study are included in the article. Further inquiries can be directed to the corresponding author.

## Abbreviations

The following abbreviations are used in this manuscript:

AFB	Acid-fast bacillus
ATP	Adenosine triphosphate
BPaLM	Bedaquiline, pretomanid, linezolid, and moxifloxacin
CRP	C-reactive protein
CT	Computed tomography
DST	Drug susceptibility test
ESR	Erythrocyte sedimentation rate
FDG	Fluorodeoxyglucose
PET	Positron emission tomography
HERZ	Isoniazid, rifampin, ethambutol, and pyrazinamide
MDR	Multidrug-resistant
MGIT	Mycobacteria growth indicator tube
MRI	Magnetic resonance imaging
OECD	Organization for Economic Co-operation and Development
PCR	Polymerase chain reaction
RR	Rifampin-resistant
TB	Tuberculosis
WBC	White blood cell
WHO	World Health Organization
XDR	Extensively drug-resistant
